# Assessing the capability to experience a 'good death': A qualitative study to directly elicit expert views on a new supportive care measure grounded in Sen's capability approach

**DOI:** 10.1371/journal.pone.0193181

**Published:** 2018-02-21

**Authors:** Philip Kinghorn, Joanna Coast

**Affiliations:** 1 Health Economics Unit, Institute of Applied Health Research, University of Birmingham, Birmingham, United Kingdom; 2 School of Social and Community Medicine, University of Bristol, Bristol, United Kingdom; University of Stirling, UNITED KINGDOM

## Abstract

**Background:**

Sen’s capability approach is underspecified; one decision left to those operationalising the approach is how to identify sets of relevant and important capabilities. Sen has suggested that lists be developed for specific policy or research objectives through a process of public reasoning and discussion. Robeyns offers further guidance in support of Sen’s position, suggesting that lists should be explicit, discussed and defended; methods be openly scrutinised; lists be considered both in terms of what is ideal and what is practical (‘generality’); and that lists be exhaustive. Here, the principles suggested by Robeyns are operationalised to facilitate external scrutiny of a list of capabilities identified for use in the evaluation of supportive end of life care.

**Methods:**

This work started with an existing list of seven capabilities (the ICECAP-SCM), identified as being necessary for a person to experience a good death. Semi-structured qualitative interviews were conducted with 20 experts in economics, psychology, ethics and palliative care, to facilitate external scrutiny of the developed list. Interviews were recorded, transcribed and analysed using constant comparison.

**Results:**

The seven capabilities were found to encompass concepts identified as important by expert stakeholders (to be exhaustive) and the measure was considered feasible for use with patients receiving care at the end of life.

**Conclusion:**

The rigorous development of lists of capabilities using both initial participatory approaches with affected population groups, and subsequent assessment by experts, strengthens their democratic basis and may encourage their use in policy contexts.

## Background

There has been increasing interest since the early 2000s in using Amartya Sen’s capability approach [1, 2] as a framework within which to evaluate health and social care [3, 4]. Much of the motivation for adopting Sen’s framework has been to capture broader (non-health) outcomes. Indeed, in the UK, the National Institute for Health and Care Excellence (NICE) has recently recommended the use of several capability based instruments, including the ICECAP-A, for the assessment of well-being in the general adult population, within its guidelines for the evaluation of social care [5]. However, Sen’s capability approach is not a fully formulated theory of social justice [6] from which measures can be generated directly. Robeyns, for example, has identified three theoretical specifications which can be thought of as decision nodes, determining the direction taken by those operationalising the capability approach: the choice between functionings and capabilities, the selection of relevant capabilities and the weighting of different capabilities (see section I in [7]). Each specification has been the subject of debate, but the most intense debate has concerned the selection of relevant capabilities [6–11].

Whilst supporting a democratic process for making these choices, Sen gives little indication of what this would entail, other than “public reasoning and discussion” (see page 77 in [11]), and there is little understanding more generally of what characterises a sufficiently democratic process [8]. Robeyns suggests a detailed procedural criteria for the selection of capabilities [7] involving four steps. First, the list should be explicit, discussed and defended. The methods used to generate the list should be clarified and scrutinized [7]. The list should be explored and discussed in terms of what is ideal and what is pragmatic, referred to by Robeyns as ‘generality’ (see “Quality criteria for selecting capabilities in [10]). Finally, the list should be exhaustive, but equally there should be no scope for reduction. Robeyns does not stipulate who should be involved at each stage.

Whilst the capabilities included in some measures of self-reported wellbeing (developed in the contexts of health, disability and social care) have been derived from Nussbaum’s list of central capabilities [12, 13] (Nussbaum’s list being the best known example of an expert led approach), they have still tended to include participatory steps, in terms of involving potential or actual service users in refining the questions. Other measures, however, have relied on in-depth qualitative work with actual or potential service users from the outset to identify those capabilities of importance [14–16] but have not been subject to the sort of expert scrutiny recommended by Robeyns.

Our contribution here relates to the examination by experts of an existing list of seven capabilities focused on the opportunity for a good death. Care at the end of life is a policy priority in most high income countries, with concerns around the increasing medicalization of death [17], commonly within a hospital setting [18, 19], with insufficient focus on care, compassion and communication [20]. Care at the end of life also represents one context in which the limitations of common health economic frameworks such as cost-utility analysis are particularly stark. The opportunity for a good death has recently been advocated as an important outcome in evaluating end of life care [21]. The list of capabilities examined was developed through in-depth qualitative work with those at various points on the end of life trajectory [22]. It has been interpreted in the form of a measure, the ICECAP-SCM (supportive care measure), grounded in Sen’s work on capability.

ICECAP-SCM was developed for use in economic evaluation. Economic evaluation can be defined as “the comparative analysis of alternative courses of action in terms of their costs and consequences” (see page 9 in [23]). In countries with a defined and centralised process for appraising potential healthcare interventions, a one-off decision tends to be made about whether an intervention should be made available within a healthcare system, based (at least in part) upon evidence from economic evaluation. Interventions approved for use in the healthcare system can then be considered as options for the treatment of individual patients, with individual treatment decisions being made on the basis of clinical judgement/guidelines and patient choice. Hence, instruments designed for capturing consequences for inclusion within economic evaluation are not typically used to inform care at the patient level, and indeed their succinct and genetic nature limits their suitability for this purpose.

The ICECAP-SCM was designed to be used alongside existing capability-based measures (such as the ICECAP-A) to capture consequences that are relevant to the specific context of supportive end of life care. Whilst the ICECAP-A collects data on general well-being (in terms of attachment, stability, achievement, enjoyment, and autonomy) [14], ICECAP-SCM can be used to collect supplementary information that is contextually relevant. There was an explicit desire to develop a concise list of capabilities because of both the vulnerable service user group who will complete the measure and the desire to weight the capabilities (a task complicated greatly if the list of capabilities is extensive). The seven capabilities included in the ICECAP-SCM relate to: choice/having a say in decision-making; love and affection/being with people who care; freedom from physical suffering; freedom from emotional suffering; dignity and self-respect; support; and preparation. Weighting provides evidence on the relative desirability of the different capabilities and avoids the need to make (unrealistic) assumptions about the linearity of the response levels for each capability. Weights for the ICECAP-SCM have been elicited through best-worst scaling [24]. There is some evidence as to the feasibility of using ICECAP-SCM with hospice patients [25], but also a need to assess feasibility in patients with distinct dying trajectories as the hospice study referred to here included mainly cancer patients. As results relating to feasibility in hospice patients were published after the interviews reported in sections two and three below, there is no chance that it will have influenced the thinking of the stakeholders that we interviewed.

If the measure is to shape policy and deliver improvements in the quality, efficiency and equity of end of life care then it must be accepted by researchers and decision-makers as feasible (pragmatic), relevant and exhaustive. Hence this paper reports work which sought to elicit the views of palliative care experts, philosophers, psychologists and economists on the list of capabilities included in the ICECAP-SCM, broadly in relation to Robeyns’ criteria of ‘generality’ and ‘exhaustion and non-reduction’. The methods used to generate the list and its content are reported elsewhere [22]. Based upon the reaction, reflections and insight of experts, the conceptual relevance and appropriateness of grounding such an instrument in the space of capability was also assessed.

The qualitative methods used to assess generality, exhaustion and non-reduction, and to give some indication of the appropriateness of the conceptual framework, are outlined in the methods section below. Exhaustion and non-reduction (whether the list truly captures what is important without attributes encompassing overlapping concepts) are reported, in turn, in the results section, followed by discussion of generality (the practical acceptability of the measure and any trade-off here with respect to the ‘ideal’), and then conceptual issues around the capability approach.

## Methods

Semi-structured interviews were conducted with academic stakeholders with views relevant to the topic of economic evaluation and end of life care. Ethical approval was obtained from the Science, Technology, Engineering and Mathematics Ethical Review Committee at the University of Birmingham [ERN_11–1293].

Relevant stakeholders (clinical and non-clinical), currently working in or visiting the UK or Republic of Ireland were identified through: searches of conference programmes; searches of public websites of key research institutions; known contacts of the research team and project advisory group; publicly listed membership of NICE Technology Appraisal Committees; as well as snowball sampling. Stakeholders were sent an invitation letter and information sheet via email; one email reminder was sent.

Semi-structured one-to-one interviews were conducted during 2012/3. Interviews lasted 45–80 minutes. Participants were presented with the list of capabilities that comprises the ICECAP-SCM in the latter part of the interview, after questioning about the purpose and current provision of end of life care and outcomes important for inclusion within economic evaluation and/or to the patient. Interviews were audio recorded and transcribed verbatim.

Interviews were conducted by PK (a health economist with previous qualitative research experience), using a topic guide developed by both authors.

Transcripts were analysed in batches of between three and six, using constant comparison [26]. For analysis, the batches of transcripts were grouped according to the academic discipline of the participant. Transcripts were read and key themes and topics identified and documented. For early interviews, two researchers (PK, JC) independently coded the transcripts, before discussing and comparing the two independent coding matrices. Themes were refined and modified as analysis progressed and descriptive accounts were formed, compared and refined.

## Results

Twenty interviews were conducted from 33 invited individuals (recruitment rate 61%) with: six health economists (HE), two philosophers/ethicists (PE), three health psychologists (HP), six nursing/allied health professionals (NA), and three physicians (DR). Participants are identified by the relevant suffix (as in brackets above) and a number, representing the order in which the interviews were conducted. Table 1 provides a summary of the academic discipline and any relevant clinical experience of those participating in the interviews.

**Table 1:** Summary of participant professional discipline & experience.

Participant ID	Discipline	Direct care/clinical experience relating to EoLC?	Policy influence?
01HP	Health Psychology	Not applicable	
02HE	Health Economics	Not applicable	Y
03NA	Nursing	Nursing (Care for the elderly)	
04HP	Health Psychology	Not applicable	
05NA	Occupational Therapy	None	
06DR	Clinical	Consultant (Palliative Medicine)	
07HE	Health Economics	Not applicable	
08NA	Nursing & Social Science	Nursing (Intensive Care & Palliative Care)	
09HE	Health Economics	Not applicable	
10NA	Nursing	Nursing (Emergency Medicine)	
11NA	Physiotherapy & Social Science	Physiotherapy (Older People’s Care)	
12HE	Health Economics	Not applicable	Y
13NA	Nursing	Nursing (Palliative Care)	Y
14DR	Clinical	GP, then Consultant (Palliative Medicine)	
15PE	Philosophy	Not applicable	
16HP	Health Psychology	Not applicable	
17PE	Ethicist	Not applicable	
18HE	Health Economist	Not applicable	
19HE	Health Economist	Not applicable	
20DR	Clinician & Charity Sector	GP	Y

Of those 13 individuals who were invited to participate but did not participate, a positive response was initially received from two (one clinician and one philosophy), but it was not possible to arrange an interview date due to their workloads. One further invitee (from an independent health policy organisation) responded to inform us that they would be spending time outside of the UK. No response was received from the remaining 10 invitees.

Because participants were asked about the purpose and provision of end of life care and outcomes of importance *before* being shown the ICECAP-SCM, responses from the first part of the interview are presented as suggestions of what *should* be included. In this way the adequacy of the final list can be assessed both through considering capabilities that experts initially listed as important and their views on the list actually incorporated within ICECAP-SCM.

### Expert views on concepts that are relevant and important at the end of life

Many participants were influenced by personal experiences and suggestions from clinical and non-clinical academics about what is important to patients were strikingly similar. Clinical academics often stressed the varied circumstances of different patients, and were more explicit about the need for care to be patient-centred. Most participants outlined three or more important outcomes, considering care in a holistic way.

…good quality pain and symptom control, psychological care, social care, and meaning making–spiritual or existential care. (01HP)

…thinking about the patient in terms of a complicated set of problems, physical, psychosocial, spiritual (08NA)

Overall, there were seven themes related to concepts that were seen by these experts as being important at the end of life.

### Communication & acknowledgement

Participants spoke about the need for good communication between health professionals and both patients and families; for example clinical academics stressed the need for health professionals to “partner with” patients and “pick up on intuitive cues”. Central to accounts of good communication was the notion that the patient is aware and can acknowledge that they are approaching the end of life, and a focus on the “open acknowledgement of dying” (06DR):

*A good death for us is that this guy maybe acknowledges and accepts*, *and we can move on to end of life care* (06DR)

*…convey to people that maybe there aren’t many more weeks and months left* (03NA)

### Emotional and psychological support, spirituality and reflection

The holistic nature of end of life care and emotional and psychological support for the patient as well as a spiritual focus were commonly discussed.

*…it’s quite intensive activity… in terms of emotional support* (20DR)

I’m sure there’s more reflection on: relationships, family, achievements… people probably become a lot more philosophical… (02HE)

Participants spoke of a need for the care environment to avoid, or at least not add to, a patient’s distress. Some also spoke of the need for clinical staff to be available and confident in interacting with patients.

*…crucial conversations happen when… someone’s lying awake at 2 o’clock in the morning… In a hospice setting… [there’s] the level of confidence in the staff… that if somebody says “I’m frightened of dying”… that member of staff can be there for an hour [offering emotional support]* (11NA)

### Choice and autonomy

Place of death and support for patients to die at home are high on the policy agenda internationally [18, 19], and so it isn’t surprising that this was mentioned by participants, many of whom are immersed in this literature. Participants recognised, however, that place of death is one among several key decisions that a patient is likely to make and that it has significance for a variety of reasons, such as the ability to be with people who are important. As 06DR illustrates in the context of the review of the Liverpool Care Pathway (a tool designed to support health professionals in planning patient care) in the UK [20], there may also be a more general fear about the loss of autonomy.

*…it’s clear that patients and families feel that their autonomy at the end of life is being taken away*. (06DR)

*He did die at home and it was what they wanted…* (11NA, referring to a personal friend)

*My whole philosophy as an occupational therapist is that care should be … client centred* (05NA)

Some participants spoke of how artificial it is to think about the patient making decisions in isolation, noting instead that they will consider the impact on and views of those close to them.

*…on an individual level*, *it’s likely that … the preferences that come across may be mediated by what the family members want… few of us are unconnected* (19HE)

Whilst participants acknowledged the difficulty of making plans, given that people have little realistic idea as to what to expect as they approach death, autonomy was still regarded as being important.

*…there are some things that*, *unquestionably*, *people know they want to avoid* (18HE)

### Being with important people

Most participants spoke about the importance of relationships with family and friends. These relationships are impacted upon by illness and circumstances in several ways, with family providing both love and psychological support, being witnesses to the patient’s death (with the pain and distress associated with this) and providing more practical informal care.

*If you go and ask patients what’s important to them… they’ll say not being a burden on their family* (14DR)

*What people need is to be in a place where … they can have their important people around them* (13NA)

### Pain and symptom control

Participants tended to refer to pain and symptom control as if they were such obvious concepts that they needed little explanation and so, whilst this theme was clearly important, it is not possible to provide a detailed exploration of the concepts.

*If you go and ask patients what’s important to them*, *they’ll say symptom control* (14DR)

*…that …the end of life is pain free* (15PE)

### Dignity and being treated with compassion

Dignity was seen as depending upon issues such as attention to personal hygiene and appropriate privacy. Comfort was often referred to alongside dignity and particularly from a nursing perspective these concepts were related to care and compassion, with 03NA referring to “tender loving care”. Care and compassion were contrasted with “heroic” medicine, which was seen as invasive, unnecessary and “toxic”.

*…she wanted to be looked after and kept comfortable* (11NA)

### Having affairs in order and having a sense of completion

This theme encompasses several quite distinct concepts or priorities, from the more practical consideration of financial affairs to a sense of resolution, completion or “a more global sense of satisfaction” (18HE). In some ways the theme is related closely to spirituality.

Again, there is awareness here of the patient’s concern for the impact upon family and friends. There is likely to be a fairly natural sequence of first sorting practical and financial arrangements and then, as death nears, “saying goodbye to relatives and making peace” (19HE).

*…but they also want a sense of completion… have some sense of resolution…* (14DR)

*what happens when I’m gone… that there’s a plan* (05NA)

*their ability to put financial affairs straight … just do those last… things that they want to do before they shuffle off*. (12HE)

### Comparing expert views on important concepts with capabilities in the ICECAP-SCM

Table 2 compares the themes identified from expert’s accounts with the attributes from the ICECAP-SCM. Acknowledgement is the only theme identified that does not directly link to an ICECAP-SCM attribute, although without some awareness and acceptance there would be limited capability in terms of some ICECAP-SCM attributes, particularly *Preparation* and *Emotional suffering*.

**Table 2:** Matching participants’ themes to capabilities listed in ICECAP-SCM.

Themes identified by participants	Capabilities listed in ICECAP-SCM
**Communication & Acknowledgement**^1^	**1) Having a say–**Your ability to influence where you would like to live or be cared for, the kind of treatment you receive, the people who care for you
**Emotional and psychological support, spirituality and reflection**	**4) Emotional suffering–**Experiencing worry or distress, feeling like a burden **6) Being supported–**Having help and support
**Choice and autonomy**	**1) Having a say–**Your ability to influence where you would like to live or be cared for, the kind of treatment you receive, the people who care for you
**Being with important people**	**2) Being with people who care about you**–Being with family, friends or caring professionals
**Pain and symptom control**	**3) Physical suffering–**Experiencing pain or physical discomfort which interferes with your daily activities
**Dignity and being treated with compassion**	**5) Dignity–**Being treated with respect, being spoken to with respect, having your religious or spiritual beliefs respected, being able to be yourself, being clean, having privacy **6) Being supported–**Having help and support
**Having affairs in order and having a sense of completion**	**7) Being prepared–**Having financial affairs in order, having your funeral planned, saying goodbye to family and friends, resolving things that are important to you, having treatment preferences in writing or making a living will

^1^ to some extent this theme is probably a prerequisite for facilitating and enabling achievement in respect to all of the capabilities listed within ICECAP-SCM

Table 3summarises the invited critique by these experts of the capabilities expressed in ICECAP-SCM.

**Table 3:** Expert reaction to attributes on ICECAP-SCM.

**1) Having a say–**Your ability to influence where you would like to live or be cared for, the kind of treatment you receive, the people who care for you
*Most*, *some*, *only a little of the time or never*. *Is having a say something that is about how often? (04HP)* *People actually want to have a view because there’s a particular thing to have a view on*, *rather than they want to have a view*. *(09HE)* *So this is about involvement in decision-making isn’t it? I think that’s really important… (19HE)*
**2) Being with people who care about you**–Being with family, friends or caring professionals
*The ‘if I want to’ about being with people is a useful addition (18HE)* *This is an important domain (14DR)*
**3) Physical suffering–**Experiencing pain or physical discomfort which interferes with your daily activities
*Emotional suffering*, *it doesn’t define what emotional suffering is… and then similarly with physical suffering*. *… I can imagine people responding to this and wanting lots of clarity about some of these things*. *(07HE)* *Suffering is a very value laden term*, *and I think that’s problematic… we’ve used very generic terms sometimes*, *like ‘concerns’ or ‘worries’ (14DR)* *I’d not have gone for suffering… suffering is a very loaded word (05NA)*
**4) Emotional suffering–**Experiencing worry or distress, feeling like a burden
*Suffering… maybe you don’t want to say this*, *or maybe you just want to say ‘worry or distress’ (13NA)* *It’s just the terminology*, *… because I work in mental health*, *it’s not really the language that we would use… (02HE)* *Experience worry or distress*, *I can understand …feeling like a burden almost feels like it would go into being supported (06DR)*
**5) Dignity–**Being treated with respect, being spoken to with respect, having your religious or spiritual beliefs respected, being able to be yourself, being clean, having privacy
*This dignity section… it’s a bit meaty [in terms of the descriptors] … I think the difficulty of that will be when they come to analyse it*. *What does dignity mean to people? (05NA)* *We did lots of work on dignity… and especially if you work with older people*, *they interpret it in very different ways… it’s great it’s in here because it’s important*, *I just wonder how easy it is for certain groups to interpret that word*. *(14DR)*
**6) Being supported–**Having help and support
*Support is a very generic term… is it help and support about health problems*, *or the needs I have at home or practical issues… I would almost be tempted to say there are different aspects of support that might need unpicking a bit (14DR)*
**7) Being prepared–**Having financial affairs in order, having your funeral planned, saying goodbye to family and friends, resolving things that are important to you, having treatment preferences in writing or making a living will
*Being prepared is great… having things in order*, *resolving things… I think that works OK (14DR)* *This is the one that stood out as being most problematic to me*, *in that … it misses out the element of getting the support I need to make those preparations (11NA)* *Being prepared is the only box that actually makes it look like an end of life care tool isn’t it? (06DR)*

Two main themes emerged from this section of the interview: use of the term “suffering” for attributes three and four; and the explicitness of the capabilities in focusing on end of life. Four participants expressed reservations about the term “suffering” (see Table 3).

There was mixed opinion about the explicitness of the instrument’s focus on end of life, with some participants expressing doubt over whether it would be appropriate to phrase attributes more explicitly.

…this is not necessarily about end of life care, it’s about something very broad, but of course they are the domains of palliative care in its broadest sense. (08NA)

Quite a lot of patients have quite a bit of denial, so it’s not right to have a very prescriptive policy where we must make everybody start talking about dying. (20DR)

There’s nothing here measuring awareness or acknowledgement about being at the end of [life] … you might risk distress I suppose (06DR)

I guess one thing it doesn’t do is face …the issue of death. I’m not sure if you’d want it to (18HE)

### Feasibility/Generality of the measure

Participants were aware that those at the end of life are often very vulnerable, and they generally welcomed the simplicity of ICECAP-SCM. There was a common view that the ICECAP-SCM could feasibly be self-completed by some patients, but equally acknowledgement that there would also be a need for proxy completion.

It’s short and it’s fairly simple (07HE)

*I wonder if for some of these you may actually have hit on aspects that carers CAN judge* (02HE)

Some participants suggested that aspects of the layout, wording and structure of the questionnaire created a degree of complexity.

…some of the [response options] relate to the current… ‘I’m able to make decisions’, it suggests that you’re talking about now… But if you say ‘I’m never able to’, it has connotations beyond the current time … it takes you back to the past…” (14DR)

Because of the way the response options repeat… it looks longer and a bigger deal to complete than it probably is (18HE)

Overall reaction to the content was positive:

… this has got a good chance of having reasonably good measurement characteristics because they’re–in general–picking up things that are important to people and expressing this in accessible language (09HE)

… if asked would I be willing to include this as an outcome measure in studies that we were doing… I’d be willing to consider it… yea, would be very willing to consider it (04HP)

So they’re all things that the literature would say exemplify a good death (06DR)

“all of these are reasonable dimensions… yeah, I think that’s rather good actually. Seems to be covering everything” (20DR)

### Evaluating outcomes in terms of capability for a good death

An important element of care at the end of life involves enabling the patient to make choices and have autonomy. In this sense a measure that has its theoretical grounding in Sen’s capability approach seemed conceptually appealing to some experts, particularly those with a clinical background.

*The goals … become much more patient-centred… when it comes to end of life care*, *you don’t know what the patient wants and so you have to negotiate with them* (06DR)

*[Care at the end of life is] about enabling people to have what they wish* (05NA)

Agency, defined by Sen as encompassing “all the goals that a person has reason to adopt, which can *inter alia* include goals other than the advancement of his or her own well-being” [1] also seemed important to the expert stakeholders, a number of whom felt that goals other than advancement of the patient’s own immediate wellbeing would be important in the context of end of life care.

*…the patient isn’t just considering their own interests but will also be interested in how their family will be supported*, *that “we’re all supported while I die”* (11NA)

*[the patient] can place greater value on …the wellbeing of other people than [they do] on [their] own state*, *be it physical or mental… that’s why we make wills for example*. (17PE)

Although agency is a potential strength of this conceptual approach, there was some uncertainty around whether the ICECAP-SCM adequately captures an individual’s goals concerning the wellbeing of others.

*There may be a little bit that’s missing from here which is about… concerns about family and friends… What was terribly important to them was … helping their family come to terms with and adapt to the idea that they would die* (08NA)

*there’s a big focus here on the individual… one of the things I’m genuinely interested in is thinking about whether there are problems in having this individualist focus within ethics* (15PE)

Two capabilities (“Emotional Suffering” and “Being Prepared”) do, however, explicitly incorporate the concept of agency, in terms of fear of being a burden on others and preparing a will.

## Discussion

This paper provides a participatory examination of a list of capabilities generated to examine the opportunities that people have for a good death. Following the work of Robeyns [10], the research provided the opportunity for experts from a number of disciplines to suggest important capabilities for those at the end of life and then to comment on an existing list; the ICECAP-SCM was scrutinised in terms of its feasibility (or generality) and scope (whether the list of capabilities is exhaustive). This is the first time such an approach has been used to consider capabilities at the end of life, and one of the first empirical explorations of the use of democratic principles in exploring lists of capabilities. The focus that stakeholders had on patient-centred care justifies the grounding of the measure in a capabilities space (for the same reasons that Entwistle [27] has presented the merits of a capabilities approach to promote patient-centred care). The research also indicated that the experts were broadly in agreement with the capability list, suggesting that it could be taken forward for use in policy evaluation. The broad concepts covered by each of the seven capabilities allow for heterogeneity in terms of personal priorities aligning with the focus on patient-centred care whilst providing a measure that can collect data at the level at which it can be used by policy and decision-makers.

One issue raised by a minority of participants was the terminology of “suffering”. Choice of the term suffering was based upon analysis of qualitative evidence from the public [22]. Sutton and Coast [22] note that at the point of determining wording for the measure, “suffering was easily understood by participants, who related this to experiencing pain, discomfort or fatigue” (p4). There is therefore, potentially, a conflict between the public and the experts here in relation to this terminology. A think-aloud study with patients, clinicians and close persons, conducted in a UK hospice setting, did not reveal concerns about use of the term “suffering” [25], but this should be closely monitored in future work exploring the feasibility and validity of the ICECAP-SCM measure.

A similar approach (seeking expert feedback) has been used in relation to one other capability measure [28], although a less comprehensive group of experts was included in the Keeley *et al* study. There was greater agreement amongst the experts in this end of life study about the list of capabilities generated than in the Keeley *et al* study, possibly because end of life is a more tightly defined period in life, where the capabilities of importance to people may be more homogeneous. We are not aware of other studies in which a direct and rigorous effort to obtain feedback on a list of capabilities has been adopted.

There are some limitations to the work. Inevitably, decisions about who to involve in the participatory process were made by the research team [8]. Participants were recruited for their expertise relating to health and social care decision making (particularly resource allocation) and care at the end of life. None had a specific research interest in the capability approach, and so there was a tendency to compare the measure and capabilities to the more generic Quality-Adjusted Life-Year (QALY) or dimensions included within condition-specific palliative care tools as a reference. Nevertheless, it was deemed important to gather evidence from those with an in-depth understanding of the particular context in which the capability list will be used, so this can also be seen as a strength of the study.

An area where further research may be required relates to the issue of agency, and given that experts have highlighted the importance of agency in terms of the patient’s consideration of others, this issue should be given attention as the ICECAP-SCM is piloted with the target population group, in terms of whether they feel that the attributes adequately express their concern for others. It may also be important to assess the capabilities of friends and relations at the end of life [21, 29].

## Conclusion

Overall the ICECAP-SCM measure was deemed to be feasible and it appears to be exhaustive in terms of its inclusion of relevant capabilities/concepts. There was some debate around the terminology used; given that terminology was informed by in-depth qualitative work with those at the end of life, this issue reveals a possible conflict in terms of whose views should be given the most weight.

The adoption of evaluative frameworks grounded in Sen’s capability approach represents a significant departure from the dominant healthcare decision making frameworks used in countries such as the UK and Australia. There is potential for capability-based approaches to be accepted in the context of end of life care because of the distinct characteristics of this particular context; just as policy organisations such as NICE in the UK have recently allowed the use of capability measures for the evaluation of social care [5, 30]. The rigorous development of lists of capabilities using both initial participatory approaches with relevant groups, and subsequent assessment of these lists of capabilities by relevant experts, strengthens the democratic basis for these lists, and enhances the likelihood of the approach influencing decision making in practice.

Further evidence on feasibility and validity of the ICECAP-SCM in particular is needed (particularly across varying trajectories towards death and in varying care settings), but there is certainly cause for optimism that the measure can appropriately be used to inform the distribution of scarce health and care resources in the area of supportive end of life care.
